# Real-Time Avoidance of Ionising Radiation Using Layered Costmaps for Mobile Robots

**DOI:** 10.3389/frobt.2022.862067

**Published:** 2022-03-17

**Authors:** Andrew West, Thomas Wright, Ioannis Tsitsimpelis, Keir Groves, Malcolm J. Joyce, Barry Lennox

**Affiliations:** ^1^ Department of Electrical and Electronic Engineering, University of Manchester, Manchester, United Kingdom; ^2^ Department of Engineering, Lancaster University, Lancaster, United Kingdom

**Keywords:** nuclear, radiation, inspection, autonomy, ROS, field robotics, ALARP, ALARA

## Abstract

Humans in hazardous environments take actions to reduce unnecessary risk, including limiting exposure to radioactive materials where ionising radiation can be a threat to human health. Robots can adopt the same approach of risk avoidance to minimise exposure to radiation, therefore limiting damage to electronics and materials. Reducing a robot’s exposure to radiation results in longer operational lifetime and better return on investment for nuclear sector stakeholders. This work achieves radiation avoidance through the use of layered costmaps, to inform path planning algorithms of this additional risk. Interpolation of radiation observations into the configuration space of the robot is accomplished using an inverse distance weighting approach. This technique was successfully demonstrated using an unmanned ground vehicle running the Robot Operating System equipped with compatible gamma radiation sensors, both in simulation and in real-world mock inspection missions, where the vehicle was exposed to radioactive materials in Lancaster University’s Neutron Laboratory. The addition of radiation avoidance functionality was shown to reduce total accumulated dose to background levels in real-world deployment and up to a factor of 10 in simulation.

## Introduction

Robots are an excellent technology for completing dull, dirty, and dangerous tasks in nuclear facilities whilst removing humans from unnecessary danger from radiological, chemical, or physical hazards. In the United Kingdom, robotics has been highlighted by the Nuclear Decommissioning Authority as a critical part of the ongoing decommissioning of legacy facilities, which represents a projected cost of £130 billion over the next 120 years (Nuclear Decommissioning Authority, 2020a). The use of robotics can reduce financial costs, accelerate adoption of key activities, and help protect human health (Nuclear Decommissioning Authority, 2020b).

Despite mobile robots already performing some routine inspection and maintenance tasks in the nuclear sector (Nuclear Decommissioning Authority, 2020b), there is an aspiration for a 50% reduction in human-led activities in hazardous environments by 2030 (Nuclear Decommissioning Authority, 2021). Therefore, the uptake of robotics needs to increase dramatically to reach these targets. Furthermore, these robots will be expected to operate in increasingly hazardous environments compared to current activities.

Though robotic systems are generally far more tolerant of ionising radiation than humans, they are still vulnerable to ongoing damage that causes component and material degradation, unexpected behaviour, mechanical faults, and eventual demise. Total ionising dose (TID), accumulated exposure over the lifetime of a component, is used as a primary indicator of component health rather than instantaneous dose rate.

Radiation tolerance must be considered as part of both robot and mission design for missions in extreme environments (Zhang et al., 2020; Yirmibeşoğlu et al., 2019; Nagatani et al., 2011; Tsiligiannis et al., 2020). An exemplary case is the failure of early robots at Chernobyl as a consequence of ionising radiation (Tsitsimpelis et al., 2019). Furthermore, information from sensors used for inspection or robot situational awareness, such as cameras (Meng et al., 2003) and range-finding sensors (Cao et al., 2012; Diggins et al., 2015), may also become degraded or compromised, rendering the data they collect unsuitable to stakeholders. As such, robot design and mission planning may need to specifically factor in the monitoring and replacement as preventative maintenance of such equipment to avoid total system failure. For reconnaissance robots deployed at Fukushima, cameras and lidar units were most likely to fail with exposure to gamma rays after 120 Gy (Nagatani et al., 2011), with computers capable of withstanding similar exposure (West et al., 2021a).

Designing robots to be more radiation tolerant is the standard approach taken to mitigate the impacts of ionising radiation. This is typically achieved through the addition of shielding materials (Zhao et al., 2020; Nagatani et al., 2011), or by replacing vulnerable components with radiation-hardened equivalents. Radiation-hardened electronic components offer higher TID performance, but can be three orders of magnitude more expensive than their commercial off-the-shelf (COTS) equivalents (Merl and Graham, 2016) and may fall short of the required computational resources used in many modern robotic and AI solutions. Autonomous behaviours such as collision avoidance, mapping, path planning, and object recognition (Tsitsimpelis et al., 2019; Schneider and Wildermuth, 2011; Groves et al., 2021) are becoming reliant on more powerful algorithms and even specialist GPU acceleration hardware, particularly for machine learning and vision processing. Furthermore, the UK nuclear sector has expressed a preference for COTS parts and components over bespoke systems to reduce cost and time to deployment and provide better technology readiness levels and evidence of continued operation (Smith et al., 2020). Therefore, use of radiation-hardened components in robots for the civil nuclear sector is unlikely for all but the most extreme environments.

Shielding with materials such as lead or tungsten can protect sensitive electronic components, limiting the TID experienced by components, but introduces additional mass (West et al., 2021a). This extra mass is not only a significant inconvenience for operators, especially in emergency response scenarios (Kawatsuma et al., 2017), but also places greater requirements on motors and power electronics, as well as reduces mission run time for battery powered systems. Furthermore, optical or range-based sensors such as cameras, lidar, radar, and ultrasound cannot be completely shielded with opaque materials.

For the reasons stated previously, a complementary mitigation strategy is required to prolong robot lifetime by reducing radiation exposure whilst not relying solely on radiation hardening or shielding. The approach developed in this work aims to reduce radiation exposure irrespective of the radiation tolerance of the robot, by providing the robot with situational awareness of the radiation field and the ability to take action based on this information. Human practices for dose mitigation can act as an inspiration for possible robotic approaches.

Humans working in hazardous environments may adopt a principle called ALARP (as low as reasonably practicable) (Baybutt, 2014) or ALARA (as low as reasonably achievable), where risk from ionising radiation and other hazards should be minimised where possible. Robots destined for nuclear environments currently do not practice ALARP. However, by enabling robots to autonomously or semi-autonomously act to minimise their radiation exposure, they can provide a greater return on investment by prolonging their operational lifetime, increasing the reliability of systems, and acquiring better-quality survey data from on-board instrumentation.

As the robot traverses an environment, if it can deliberately take action to avoid regions of increased ionising radiation, then the overall accumulated dose for the same required task will be reduced. By avoiding ionising radiation to begin with, the TID requirements over the robot lifetime can be reduced, requiring less shielding mass or removing the need for specific radiation-hardened devices. If mobile robots can avoid damage from ionising radiation, the more attractive the use of affordable COTS hardware becomes, enabling the uptake and deployment of robotics to accelerate in these hazardous environments. These systems can be lighter, longer-lasting, more affordable, and more reliable during autonomous tasks than previous solutions.

To achieve awareness and avoidance based on radiation intensity, this work proposes the use of layered costmaps to provide additional information to path planning when radiation hazards exist. Path planning algorithms then penalise navigation through regions of increased radiation, preferring less-radiation-intensive routes, possibly at the expense of longer path lengths. The accumulated dose for a robot is, therefore, reduced with every mission, prolonging its total lifetime and that of its accompanying sensors. Moreover, this work provides a method to adapt point radiation observations, from on-board radiation monitoring instruments, into broader robot configuration space relevant information for a layered costmap from sparse, noisy, low-spatiotemporal-resolution data. This solution is demonstrated both in simulation and using a mobile robot exposed to a Cf-252 neutron source, with the method incorporated into the Robot Operating System (ROS).

## Materials and Methods

Previous literature on minimisation or avoidance of radiation exposure has been understandably focused on humans walking through nuclear environments (Pachter and Pachter, 2001; Khasawneh et al., 2013; Liu et al., 2014, Liu et al., 2016; Chao et al., 2018; Wang and Cai, 2018). In emergency scenarios, quick path planning can guide radiation workers to a point of egress whilst minimising dose. Primarily, investigations have trialled different path planning algorithms such as Dijkstra, A*, and RRT* to increase the speed at which a solution is found whilst assessing the compromises made in total dose along the path if non-optimal (Adibeli et al., 2021). This use case is intended for time-critical one-off emergency response scenarios rather than the continuous re-planning that modern mobile robots employ.

These human-centric previous works focus on the minimisation of radiation dose only, with some including binary obstacles (Liu et al., 2016; Chao et al., 2018; Wang and Cai, 2018), and are, therefore, incapable of incorporating other risks, including dynamic obstacles such as humans and including optimisation regarding path length. Furthermore, they all assume total *a priori* knowledge of the radiation field in an environment with mostly holonomic human agents, which does not hold for all robots.

Robots being deployed into radioactive environments are often the first witnesses of conditions inside facilities (Tsitsimpelis et al., 2019; Nagatani et al., 2013). Therefore, there is no prior knowledge of the radiation fields inside; ergo, previous methods are unsuitable without modification. Robots need to be able to react in real time to new information from radiation sensors and make updated path planning decisions with incomplete knowledge. Moreover, mobile robots lack the dexterity and improvisation skills of humans when traversing cluttered, unstructured environments. A priority for a mobile robotic system may not be necessarily dose minimisation, but safe and reliable traversal of otherwise challenging terrain without contacting obstacles, which may include humans. Path minimisation on dose rate alone could be potentially dangerous to robotic systems and their mission objectives.

For modern robotic platforms leveraging ROS, operators have a variety of global and local path planners at their disposal. From the standard ROS navigation stack, it is possible to choose from common Dijkstra and A* approaches, amongst others, as global planners, with a variety of local planners. Critically, these path planners are often chosen due to the physical specifications of a robot platform and its locomotion, rather than their speed of computation. An example would be a planner chosen for Ackermann steering, a non-holonomic system. Therefore previous works which are based around the holonomic movement of humans may not necessarily transfer. Layered costmaps are largely planner agnostic and could be used with most of the human-specific path planning algorithms employed in previous work. They allow for the inclusion of a variety of risk vectors, including static and dynamic obstacles, and also behavioural modifications such as no-go zones, providing humans with additional personal space, or travelling on a particular side of a corridor (Lu et al., 2014). This work also provides a method for a costmap to be updated in a robot-contextualised manner in real time.

Costmaps, an evolution of occupancy grids, are a common approach to monitoring obstacles and other hazards for path planning and avoidance. A metric map of an environment is either provided or created using SLAM (Simultaneous Localisation And Mapping), based on a regular grid of cells of set physical size, effectively discretising an environment into a downsampled finite representation. This is most commonly performed in two dimensions; however, it can be extended into higher dimensions using voxels.

The value in each cell is a representation of additional risk associated with environmental features located in that physical space. Path planning algorithms can, therefore, calculate grid-based optimised paths whilst accounting for environmental features. Costmaps allow for scalable assessment of risk over earlier binary/trinary occupancy grids, greatly increasing the granularity with which systems can plan paths around environmental features. An exemplar of costmap granularity is the inflation of obstacles into the configuration space of a robotic system, including a decreasing function to provide additional clearance and smoothing of manoeuvres through congested spaces (Zheng, 2017). The costmap implementation used in this work is handled by the ROS *costmap_2d* ^1^ package, allowing simple integration into ROS path planning and navigation handlers such as *move_base* ^2^.

Path planning occurs at two levels, global and local. Global path planning handles optimisation of paths between two waypoints and is calculated relatively infrequently along the total path, often on a coarser resolution costmap. Local path planning then acts to keep the robot on that optimised trajectory whilst responding to new information, for example, transient obstacles such as people or where obstacles are unexpected, such as furniture being re-positioned. In ROS, two costmaps can exist for each respective path planner: a global costmap that can increase in size to accommodate new locations and store previous information and a local costmap that is of fixed size around the centre of the robot. For a robot to employ this navigation system and avoid ionising radiation, it must be capable of providing costmaps at a local and global level.

The implementation proposed in this work produces a metric costmap based on the risk associated with ionising radiation, suitable for many robot designs and locomotion schemes. This can be used exclusively or with other information, including obstacles and preexisting occupancy grids maps, at both a local and global level.

### Layered Costmaps

Layered costmaps allow for the holistic inclusion of multiple data types and sources. They consist of individual layers of costmaps, which are combined to produce a single monolithic representation of hazards in an environment to be passed to a navigation implementation (Lu et al., 2014). An obvious benefit to layered costmaps is the simple and, more importantly, flexible inclusion of various behaviours or sources of hazard, which only need to be updated in their respective layer. Use of layered costmaps is supported in the standard installation of ROS, and this work provides a specialised plugin for the *costmap_2d* package.

**FIGURE 1:**
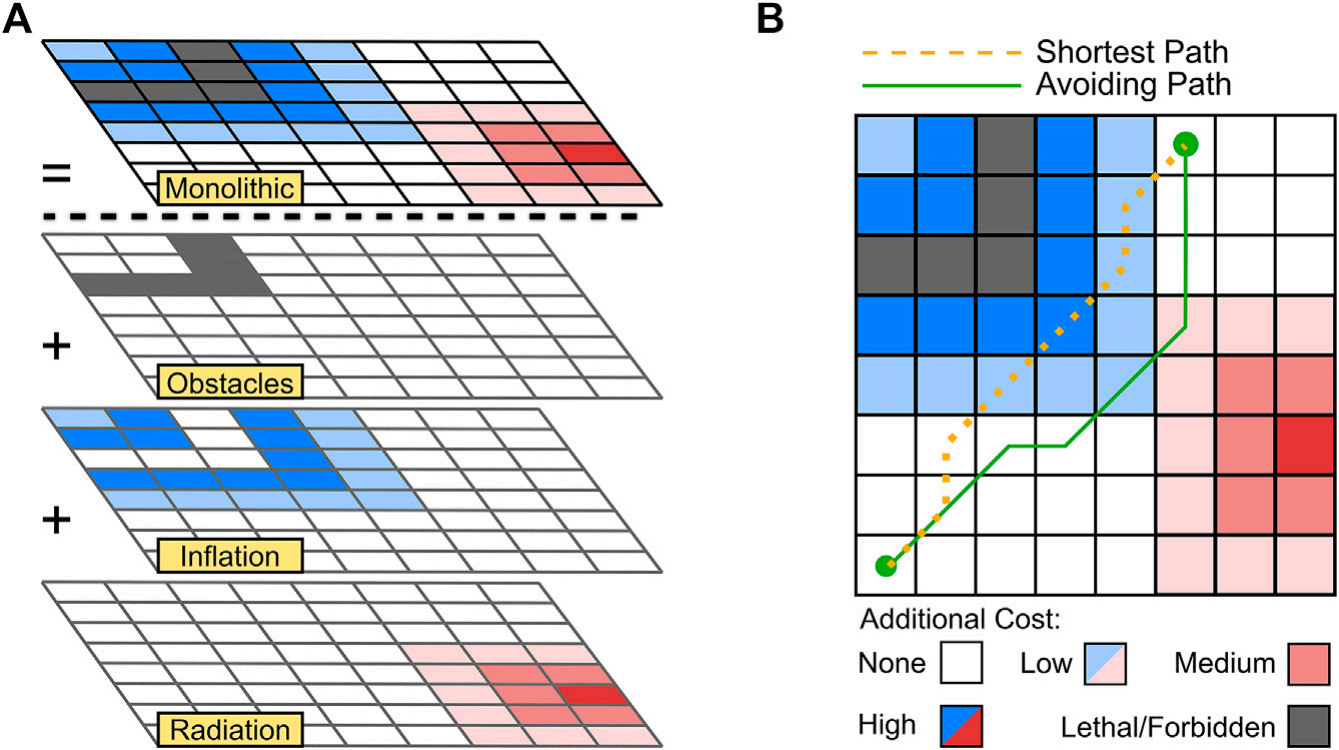
Monolithic costmap used for path planning is generated with combined information from multiple layers **(A)**. Inclusion of additional cost due to environmental risk may alter the optimal path **(B)**.

Layered costmaps have been used to great effect to modify mobile robot behaviour in social interactions, ensuring that humans are afforded a reasonable amount of personal space, navigate around moving people or crowds, and maintain social conventions (Lu et al., 2014; Kollmitz et al., 2015; Fang et al., 2020). The layers in a costmap are arranged in a hierarchy, which are then combined to produce the flattened monolithic costmap. Figure 1 demonstrates how the hierarchy of layers may dictate the behaviour of other layers and their combinations. Colours represent different layers, with darker colours indicating higher costs, lighter colours indicating lower costs, and white being indicative of no additional cost. This monolithic costmap is then used for path planning optimisation, incorporating risk from all relevant environmental factors.

The output monolithic costmap, used by the navigation stack, consists of three layers in this example. The first is an obstacle layer, referring to data from lidar, depth cameras, etc., to identify solid, possibly moving, objects in the vicinity. To convert into the configuration space of a robot, the inflation layer adds a region of increased cost around lethal (could be collided with) obstacles, including a decaying component. The radiation layer acts independently; however, to update the cost of the monolithic costmap on a per cell basis, a combination policy must be chosen.

There are typically three modes for combining layers: overwriting all other values, maintaining the maximum value, or simple addition of cost on a cell-by-cell basis. If the radiation layer simply overwrites other layers, then critical navigation features such as obstacles may be removed. To reiterate the previous discussion, obstacle avoidance and manoeuvrability of the mobile robot should be a primary consideration. Therefore, in this instance, radiation information is more suited to addition or maintaining maximum values. In this work, a map provided by SLAM and an obstacle inflation layer are used to facilitate basic navigation and path planning in conjunction with radiation awareness.

### Cost Allocation for Ionising Radiation

Path planning algorithms for point-to-point navigation are typically centred around minimising the distance travelled, to provide an optimised route from start to goal. In a discretised metric description, as is the case with costmaps, distance is represented by assigning a cost to visiting a particular cell in a costmap. To disincentivise visiting certain cells, for example, because they are close to an obstacle, an additional cost is given, mimicking a longer path length. In the costmap_2d implementation for layered costmaps, both the monolithic and individual layers are limited to integer values ranging from 0 to 255 in each cell. The values 1–252 represent a linear progression of cost, with 0 representing free space, i.e., no additional cost. For cells with no information, the value 255 is reserved; furthermore, values 254 and 253 are reserved for obstacles and circumscribed obstacles (inflation), respectively (Zheng, 2017).

For the ROS 2D navigation stack, the cost of visiting a costmap cell, *G*_*i*_, is given by

$$G_{i}=\alpha c_{i}+n,$$ (1)

where *α* is a cost scaling factor, *n* is the neutral cost, and *c*_*i*_ represents an additional cost between 0 and 252. When no additional risks are present, the cost of visiting a cell in free space (*c*_*i*_ = 0) is set by only the neutral cost. Therefore, minimisation of cost also minimises the number of cells visited, achieving the shortest path. The total path cost *P* for a given path is given by

$$P=\alpha \underset{i}{\sum }c_{i}+np.$$ (2)

Once again, in the case that no additional costs are present (i.e., *∑*_*i*_ *c*_*i*_ = 0), the optimal path will minimise the total neutral cost, *n*, multiplied by the path length, *p*, and, hence, the distance travelled. Additional costs, e.g., in close proximity to an obstacle, mimic additional costs due to a longer candidate path and are, therefore, undesirable and, hence, avoided. This model of cost can be directly mirrored to radiation dose on a per cell visited basis and motivates the use of costmaps for describing ionising radiation risk. Assuming a robot travels at a fixed velocity, it is assumed the time taken to traverse a cell will be constant. Therefore, exposed dose as a function of time is directly proportional to path length and the number of cells traversed.

The low levels of background ionising radiation dose are not treated as a concern under usual operation; therefore, regardless of robot motion, there is an equivalent neutral dose (analogous to neutral cost) associated with travel in general, even with no additional nuclear materials. The concept of a neutral dose rate is in alignment with ALARP principles, where “broadly acceptable risks” are not subject to additional mitigating actions (Baybutt, 2014). Accumulated dose can be modelled as an analogous neutral cost due to background levels, with the risk of additional dose from sources in an environment being modeled as an additional cost. Along a given path length *p*, background neutral dose rate *b*, and cell-wise additional dose *v*_*i*_, accumulated dose *D* along a path is given by

$$D=\underset{i}{\sum }v_{i}+bp.$$ (3)

By comparing Eq. 2 with Eq. 3, it is possible to create a direct relationship between additional radiation dose and the integer values of additional cost in a costmap by imposing a condition where additional radiation exposure is treated as analogous to an increase in path length. As stated previously, the total ionising dose is of greater importance to component integrity than instantaneous dose rate; hence, minimisation of integrated dose along the path is considered in this approach. Though path lengths may be longer with radiation-aware planning, the total dose will be reduced.

As an example, the hypothetical shortest path, *p*_0_, passes cells with an additional dose above the neutral background dose rate, whilst another possible path, *p*_1_, is longer but does not pass any cells with additional dose. If the extra neutral dose of the longer path is less than the additional source dose of the shortest path, i.e., *b*(*p*_1_ − *p*_0_) < *∑*_*i*_ *v*_*i*_, then the lower-dose path is preferred despite being longer. With appropriate values for *v* and *b*, the lowest-cost path should normally be the lowest-dose path, but may have considerably longer path lengths.

Radiation dose rate is on a continuous scale and may be measured in a variety of units and time scales, such as counts per second (cps) or Sieverts per hour (Sv/h). These values obtained by using instruments need to be converted to a 0–252 representation of additional cost to be integrated as part of a costmap layer. For radiation dose value *v*, the cell cost is given by

$$C_{i}=\left\lfloor 252\times \frac{v_{i}-t_{l}}{t_{u}-t_{l}}\right\rceil .$$ (4)

The lower threshold *t*_*l*_ allows for observations below this threshold to be regarded as free space, i.e., neutral dose with no additional cost, defining an acceptable background rate, or a rate which would not greatly impact the operational lifetime of the robot. The upper threshold *t*_*u*_ ensures that all readings above a certain threshold are marked as having the maximum additional cost. This allows for a linear ALARP region of dose to be defined, with dose rates above this classed as an unacceptable risk. This implementation does allow the user to set the scale factor to 252 (non-lethal) or 254 (lethal) in Eq. 4, meaning that regions of elevated radiation can be completely excluded as they are classed as lethal obstacles. This is generally not recommended, as it can have unintended negative consequences for path planning if a route becomes impassable as a result, for example, a robot being stuck behind a virtual wall of radiation and unable to return to base.

The neutral cost, *n*, and cost scaling factor, *α*, in Eq. 1 are usually chosen by a user to generate desired path planning properties or behaviours (Zheng, 2017; Lu et al., 2013). Obstacle avoidance and operator management are typically greater concerns for the operational reliability of mobile robots than radiation. The majority of robot failures in general disaster response are due to collisions, mobility failures, getting stuck, or tethers becoming severed or damaged (Murphy, 2014). Therefore, these factors should be considered first for other costmap layers such as obstacles and inflation layers. As the cost in a layer is limited to 0–255, the cost of visiting a cell *G*_*i*_ is also capped between 0 and 255; therefore, as a general rule, it is necessary to keep the neutral cost per cell low, so as to enable finer fidelity due to additional cost, rather than a planner being dominated by minimising path length.

### Interpolation Into Robot Configuration Space

For effective path planning, a planner must be able to predict conditions in the future along a path. This is straightforward for obstacle avoidance with the use of range sensors such as lidar, depth cameras, radar, or ultrasonic ranging sensors. Not only are future obstacles predictable but also the range of these sensors is much greater than the configuration space of the robot, affording information for a costmap in a whole robot’s relevant context. Radiation measurements in comparison are typically point measurements made within or in close proximity to the configuration space of a mobile robot, offering no additional future spatiotemporal information.

Point radiation measurements, therefore, require inflation to length scales similar to the configuration space of a robot platform to ensure it can correctly avoid an enlarged region around a radiation observation. Inflation of binary obstacles and a given robot length scale is trivial and performed routinely, but for radiation, it must contend with fluctuating values of radiation intensity and adequately account for gradients that may exist, particularly for gamma radiation.

To predict a radiation intensity value at the location of cell centres that do not have a direct measurement, an inference must be made from observations in the vicinity. Though there are many interpolation methods available, an approach is needed that can incorporate irregularly spaced observations as the robot moves around the environment, including repeat measurements if the robot is stationary. As radiation measurements naturally fluctuate due to the stochastic nature of radioactive decay, they must also be able to handle observations with associated variance and finally be quick to compute.

The method of inverse distance weighting was chosen for interpolation, which relies on the principle that locations close to each other are more highly correlated than distant locations, and by using surrounding observations, a value at a new location can be inferred. The use of an inverse distance weighting approach has benefits over other approaches in that it can handle fluctuating, sparse, and repeat measurements, which many interpolation techniques cannot (West et al., 2021b), but is considerably quicker to compute than more accurate methods such as Gaussian process regression.

Inverse distance weighting interpolation is performed by taking the arithmetic mean of observations, with a weighting based on how close observations are to the interpolated position. The interpolated positions in this case are the centres of each costmap cell. The spatially weighted averaged value for a given cell, *v*, for all observations, *θ*, and weighting, *w*, is given by

$$v=\frac{\sum \left(\theta_{i}\times w_{i}\right)}{\sum w_{i}}.$$ (5)

Under the assumption that observations closer to a cell are more likely to describe the conditions in that cell, observations that are closer have greater weighting than those further away. The weighting assigned is based on a Gaussian expression, given as follows:

$$w_{i}=\exp\left(-\frac{d_{i}^{2}}{2s^{2}}\right).$$ (6)

The weighting given to a particular observation, *w*, is a function of the Euclidean distance, *d*, between the observation and a given cell centre and a scale factor, *s*, which is user defined. As very distant observations are not expected to represent the conditions in a cell, observations made outside a defined region of influence are not included in the interpolation to save on computational requirements. This region can be expressed as a simple radius or as a specific polygon.

It would be inefficient for both computation speed and memory use to store all observation position and intensity values to recalculate the average of every cell upon each new observation if using Eq. 5 directly; instead, upon a new observation, only cells within a region of interest are updated given the procedure in Algorithm 1. This approach not only allows for interpolation of values to inflate radiation values into the configuration space of the robot but also acts to average inherently randomly fluctuating radiation observations, whilst maintaining gradients in the measured radiation field.

Algorithm 1: Radiation Layer Update Routine

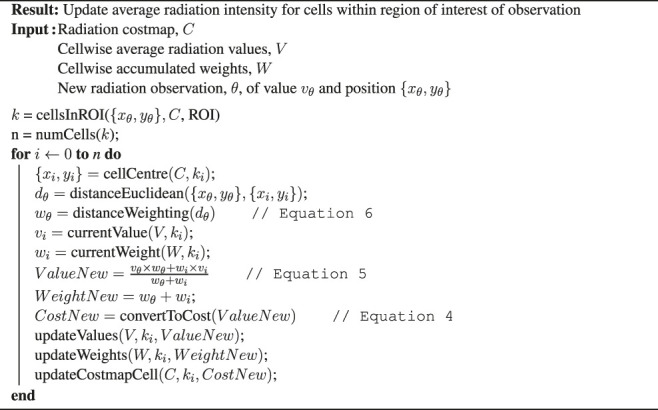

For each cell in the costmap, there are two equivalent cells held in arrays of identical size to the costmap: the one which holds the average radiation intensity value, denoted by *V*, and the one which holds the accumulative weighting of all observations that updated that cell, denoted by *W*. The cell is updated via an arithmetic mean with the previous value and weighting and the value and weighting of the new observation. The calculated average value is then finally converted to a cost using Eq. 4, and the costmap layer is updated. By storing cell-wise average radiation values, *V*, it is possible to change intensity thresholds and, therefore, cost, on an ad hoc basis.

## Results

With the advent of recently benchmarked ROS-compatible radiation simulation in the physics simulator Gazebo (Wright et al., 2021), this work uses gamma sources with a single uncollimated detector to assess the methodology before testing in an active environment at the Lancaster University Neutron Laboratory. Access to suitable ionising radiation sources can be prohibitive for research experimentation due to the availability of facilities and safety issues. Previous studies have used proxies for radiation sources, such as radio emitters (Groves et al., 2021). However, in the current work, experiments are performed in both simulation and in an active environment with real ionising radiation sources. The robot modelled in simulation is a Clearpath Jackal, reflecting the hardware used in active deployments (Bird et al., 2018; West et al., 2021b; Tsitsimpelis et al., 2021), equipped with a single front-facing lidar sensor, utilising the *GMapping* ^3^ package for SLAM. The robot uses a four-wheel skid-steer drive for forward/reverse locomotion and yaw control.

### Single Gamma Source

**FIGURE 2:**
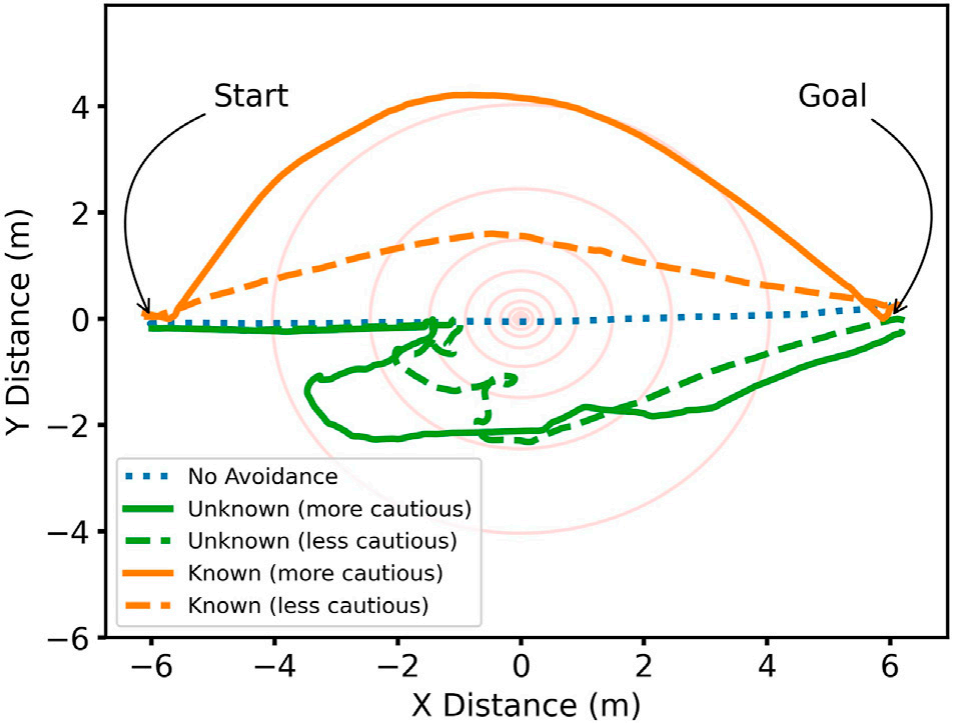
Path taken by the robot from *x* = −6 m to *x* = +6 m; when the robot conducts path planning with radiation awareness, it can avoid the gamma source located at the origin. Red circles are a visual aid of different radii from the source.

Figure 2 shows the path taken by the simulated robot in response to a single gamma source located at the origin in an open environment. The source strength at 1 m was 250 arbitrary units per second, with a neutral cell cost of 10 units, an interpolation radius of 0.5 m, and a scale factor of 0.15 m, with the lower radiation costmap threshold at 10 units and the upper threshold set at 50 or 100 units to be more or less cautious of radiation exposure, respectively. The robot was commanded to traverse from the starting position at *x* = −6 m to the goal position at *x* = +6 m, with different levels of knowledge regarding radiation. When radiation information was not included, the robot took a straight path towards the goal position and passed directly over the source. For an unknown radiation field, the robot begins to approach the source before initiating avoidance manoeuvres when the cost of continuing is too great, whereas for a known radiation field, the robot clearly takes a wider arc around the source from the beginning. When cost scaling is set so that lower intensities of radiation result in larger cell costs (upper threshold of 50), labelled as “more cautious” in Figure 2, for both the known and unknown radiation avoidance scenarios, the robot keeps a greater distance from the source.

**FIGURE 3:**
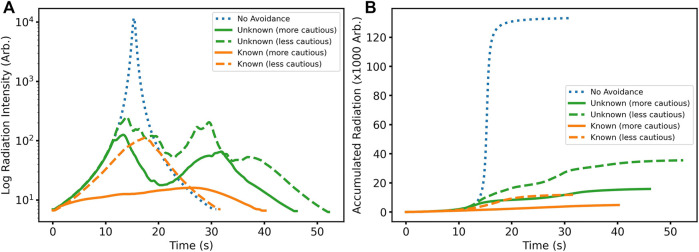
**(A)** Radiation dose rate as a function of time; instantaneous dose rate is lower when the robot is radiation aware; however, paths can take longer to complete. **(B)** Accumulated radiation dose as a function of time; total dose is decreased when the robot is radiation aware.

Figure 3A shows the clear increase in dose rate as the robot approaches the source when not avoiding radiation. It further shows an identical initial trend in dose rate for the unknown cases until the robot takes action when the dose rate becomes too high. By adjusting the threshold values, this can be triggered earlier or later depending on the radiation tolerance requirements of a robot platform, as evidenced by the earlier response of the cautious thresholding.

The resultant total absorbed dose for the task is shown in Figure 3B, calculated as the integrated dose rate over time. A considerable reduction in accumulated dose is conferred when radiation awareness is enabled, including unknown radiation fields. For known radiation environments, the accumulated dose is greatly reduced, but with a trade off of increased time to complete the task as the robot is more cautious, therefore taking a more circuitous route. As the optimisation of cost is directly related to the minimisation of dose, despite longer path lengths, total dose should be minimised over all allowable routes. When navigating a radiation environment, even in an unknown condition, with an appropriately cautious upper threshold, the total dose can be readily reduced by an order of magnitude compared to the worst case.

A disadvantage of a more cautious cost thresholding scheme is the time taken to make avoidance manoeuvres. If the robot can avoid the very central region where the dose rate is extremely high, due to the 1/*r* ^2^ relationship of intensity with distance from a point isotropic source, even mild radiation avoidance behaviours can deliver a considerable reduction in accumulated dose. Therefore, heavily penalising radiation dose is not necessary for all applications. Furthermore, this better maintains the preference for planning around other environmental features such as obstacles that pose a more immediate threat. Finally, less-cautious thresholding will result in more direct paths, making better use of other limited resources such as operator time and battery capacity.

The compromise between time taken and dose can be updated in real time in this approach by altering thresholds at any time, either by the operator or autonomous agent decision making. This could be based on the internal state of the robot, e.g., when the battery is low, the robot may prioritise more direct paths or avoid radiation more after an increased dose (Wright, 2018), or to enable greater trust for end-users who typically dislike robots behaving with higher risk in nuclear environments (Bridgwater et al., 2020).

### Autonomous Exploration With Multiple Sources

To demonstrate how radiation avoidance may be used in a deployment scenario, a simulated robot was instructed to perform frontier exploration of an environment with radiation sources located throughout the space. This was performed 20 times, both for simple obstacle avoidance navigation and obstacle avoidance navigation including radiation awareness. The end point of an exploration session was used as the starting location for the next round, to provide varied routes through the environment. For all cases, the radiation environment was unknown along with the spatial layout, so the robot was acting based on entirely new information during each exploration. This real-time response and ability to adapt may be critical for initial inspection missions in poorly documented environments.

The radiation sensor was installed at the front left corner of the chassis, but did not extend beyond the footprint of the robot. Five sources were placed in the environment, all of the 300 arbitrary units’ intensity at 1 m, with lower and upper radiation cost threshold values of 50 and 250, respectively. The interpolation distance was 1.0 m with a smoothing scale factor of 0.15 m. The frontier exploration capability was provided by the Explore Lite ROS package (Hörner, 2016).

**FIGURE 4:**
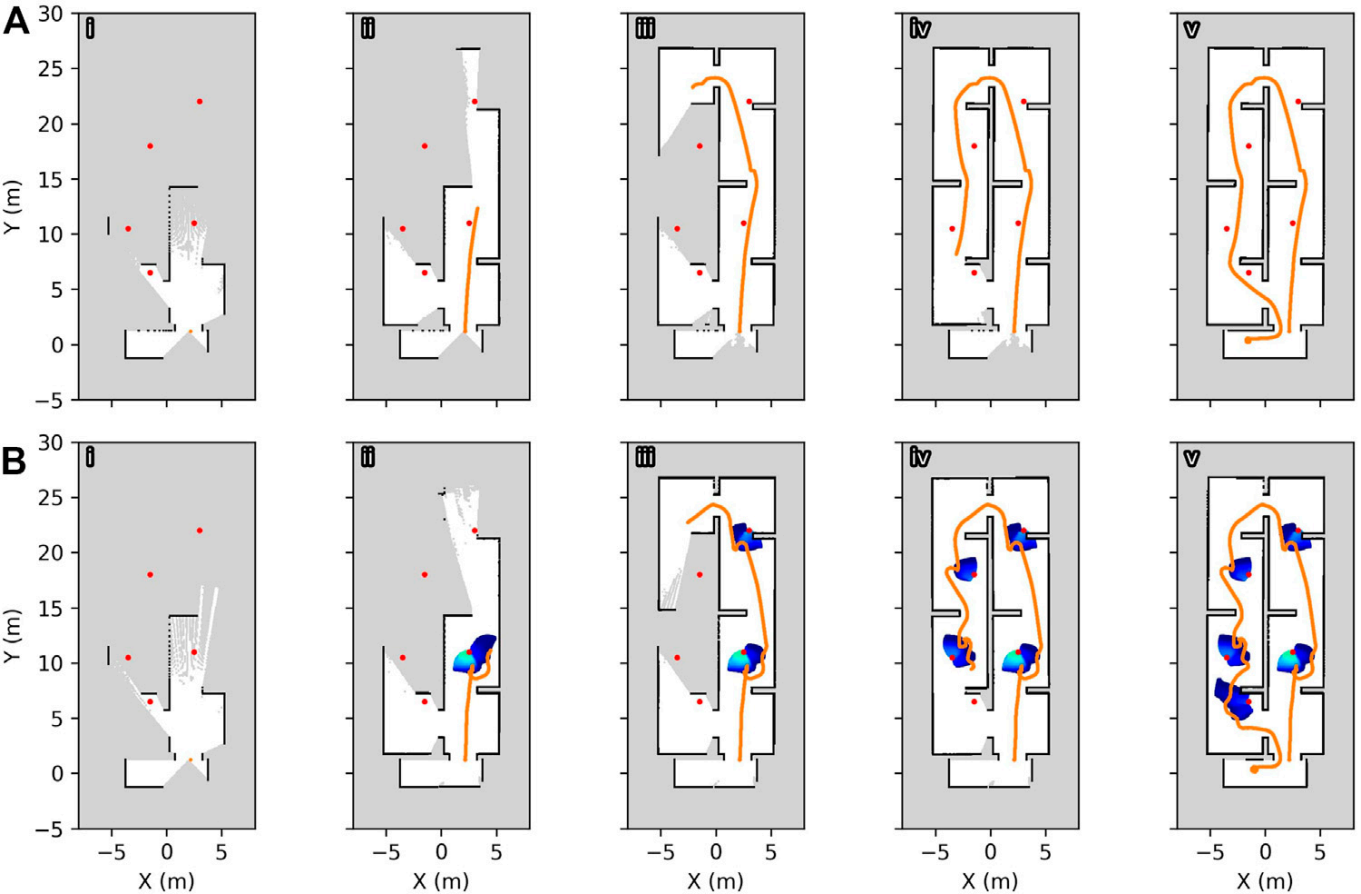
Paths taken by a simulated robot during autonomous exploration of an unknown environment, with no radiation awareness **(A)** and radiation awareness **(B)**, superimposed on the SLAM map generated during the trial, with radiation sources indicated with red markers. Snapshots are reported (i–v) from start to finish, and the path taken by the robot is highlighted. The additional interpolated cost due to radiation during exploration is expressed from blue (lowest threshold) to yellow (higher cost). The robot takes a circuitous path to avoid radiation sources when radiation awareness is enabled.

Figure 4 shows the generated SLAM maps of the environment, with example paths taken by the robot for both radiation-aware and traditional navigation. Under both schemes, the robot is capable of completing the exploration task, but when using radiation avoidance, the paths deliberately circumvent passing in close proximity to sources whilst continuing to goal locations. Coloured regions indicate elevated regions of additional cost for radiation sources at the end of exploration, with blue indicating low cost through green and yellow indicating higher cost. As the robot approaches a source, the costmap is populated with additional costs and the path planner produces an alternative route. This re-planning is performed regularly, allowing the robot to circumvent the source whilst attempting to reach an exploration target.

**FIGURE 5:**
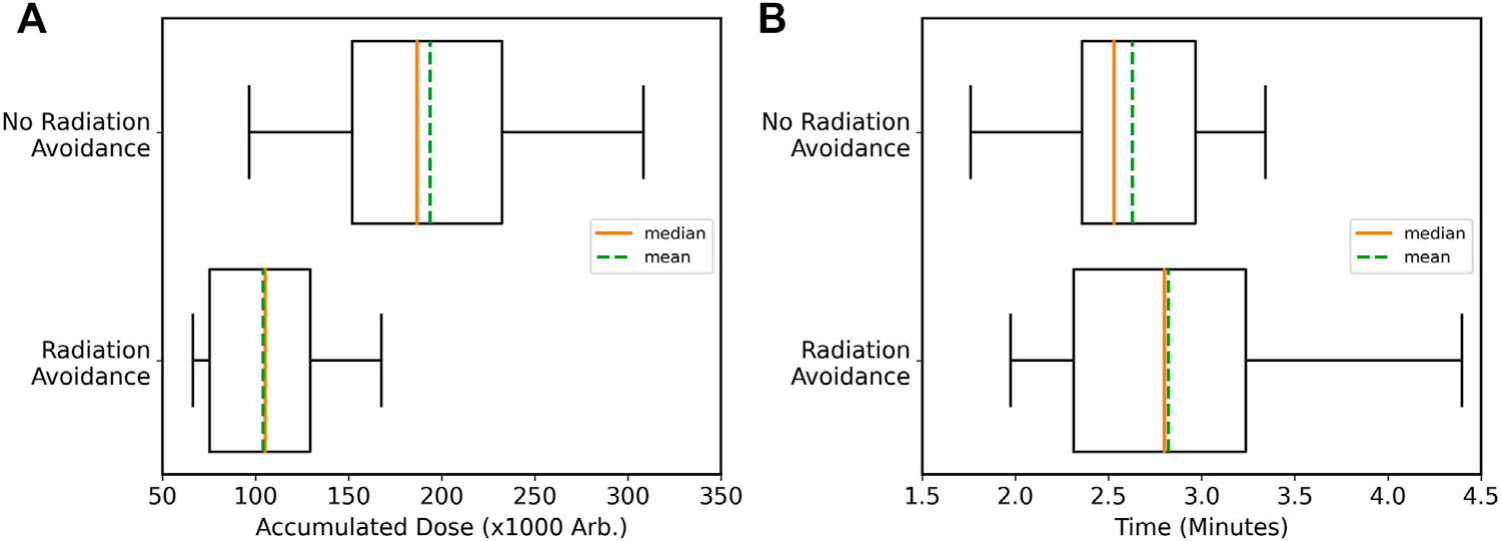
Results from autonomous exploration trials of an unknown environment. **(A)** Accumulated dose is reduced on average when using radiation-aware navigation; **(B)** an increased time to complete tasks due to longer path lengths when avoiding radiation.

The total dose received by the robot when including radiation avoidance was reduced by approximately half on average, as shown in Figure 5A for the environment and thresholding scheme used in these trials. However, due to the longer path lengths required to avoid traversing close to sources, the time to complete the exploration did increase on average, as seen in Figure 5B. As discussed previously, by manipulating the costmap thresholds, the compromise between increased time and radiation dose can be tuned and can even be altered in real time based on the robot’s internal state or preferences of the end-user.

### Lancaster University Neutron Laboratory

**FIGURE 6:**
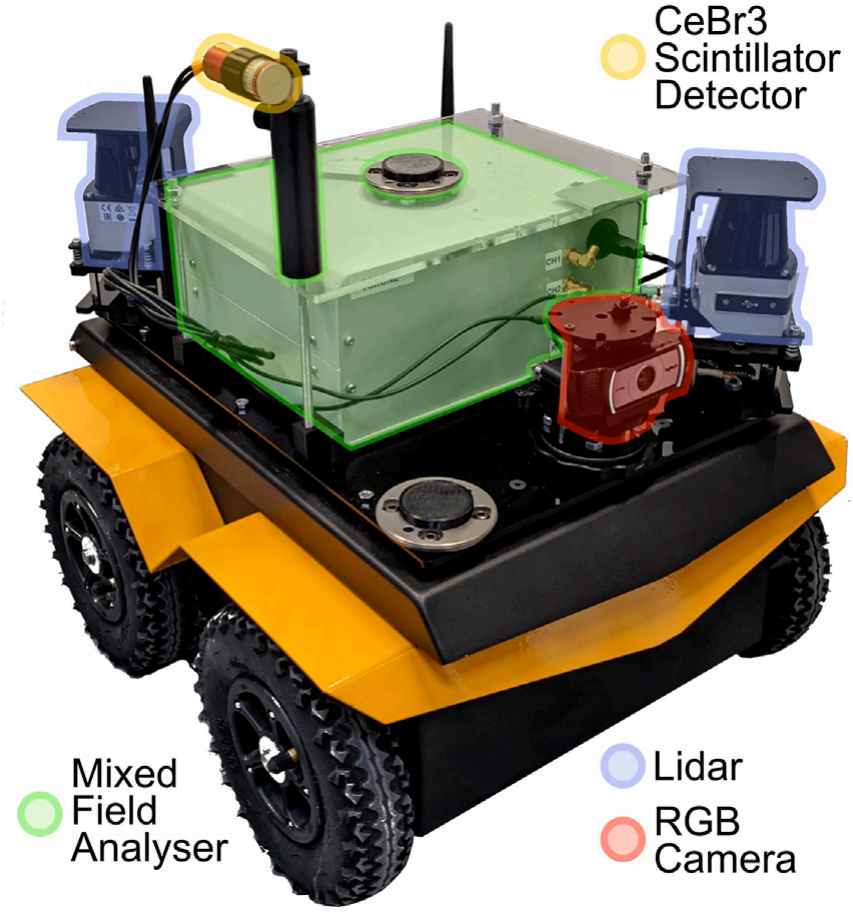
Clearpath Jackal platform, equipped with dual 2D lidar for SLAM, an RGB camera for operator awareness, and a mixed field analyser and scintillator detector for gamma ray detection.

To assess the use of costmaps to avoid radiation from real sources, a Clearpath Jackal UGV (Unmanned Ground Vehicle) was equipped with radiation sensing capabilities and deployed at the Lancaster University Neutron Laboratory, Lancaster, United Kingdom. The radiation sensor consisted of a CeBr_3_ scintillator detector, twinned with a mixed field analyser for event analysis. The count rate was reported to the robot via ROS at a rate of 1 Hz, integrated over an energy range of 300–2,500 keV. More details regarding the platform and radiation sensing can be found in the work of Tsitsimpelis et al. (2021) and West et al. (2021b). An annotated picture of the deployed platform is shown in Figures 6, 2D. Lidar (SICK TiM571) was used for SLAM, and an RGB webcam (Logitech C930) was used for operator awareness during initial manual navigation.

**FIGURE 7:**
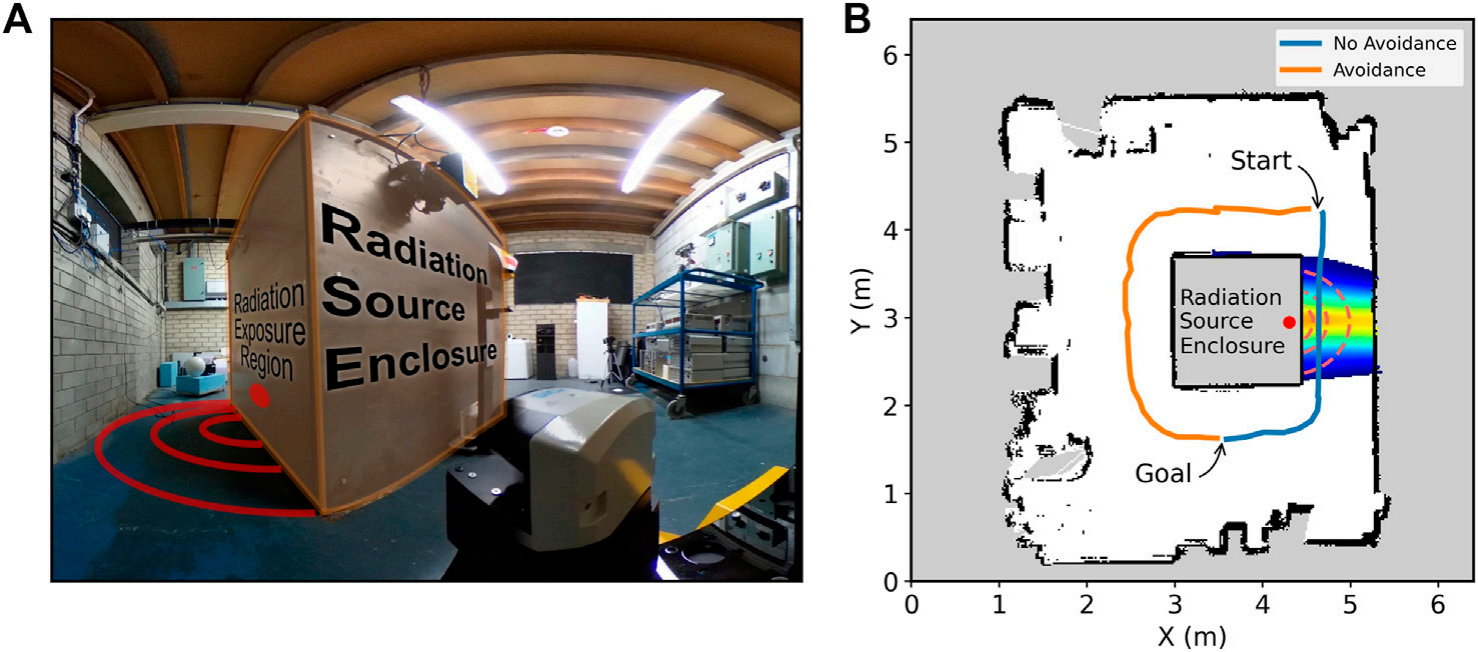
Annotated photograph taken by the robot at the starting position in the Lancaster University Neutron Laboratory **(A)**; paths planned by the robot in the presence of a Cf-252 radiation field with avoidance enabled or disabled **(B)**. Interpolated radiation field from a single pass is shown, indicating with blue (lowest threshold) to orange (high cost) increased cost to pass in front of the source.

The facility consists of a cuboid shielding container at roughly the center of the space. A 13 MBq Cf-252 neutron source is housed in a container surrounded mostly by water, and when operational, it is exposed on one side of the container, leading to a segment of the space being subject to a flux of neutrons and gamma rays. Figure 7A shows a photograph of the container at the facility and the side where the source is exposed. The shielding of the container helps to limit radiation exposure around the other three sides. Therefore, paths around these non-exposed sides offer safer routes.

Before autonomous navigation was enabled, the robot was driven manually around the environment from a remote position outside the laboratory to build up a SLAM map and also to sample the radiation field in front of the radiation source. Therefore, in this experiment, the robot is operating in a known radiation environment. The robot was then requested to traverse to a goal position autonomously, where the shortest path would take it past the exposed radiation source. Figure 7B shows a SLAM map generated by the robot, with planned paths when the radiation costmap layer was enabled or disabled, with the approximate location of the radiation source highlighted. With radiation-aware navigation, it is clear the robot has a preference for taking a longer path with a lower accumulated dose, whereas for the shortest path, the robot is subject to radiation exposure in close proximity to the source.

**FIGURE 8:**
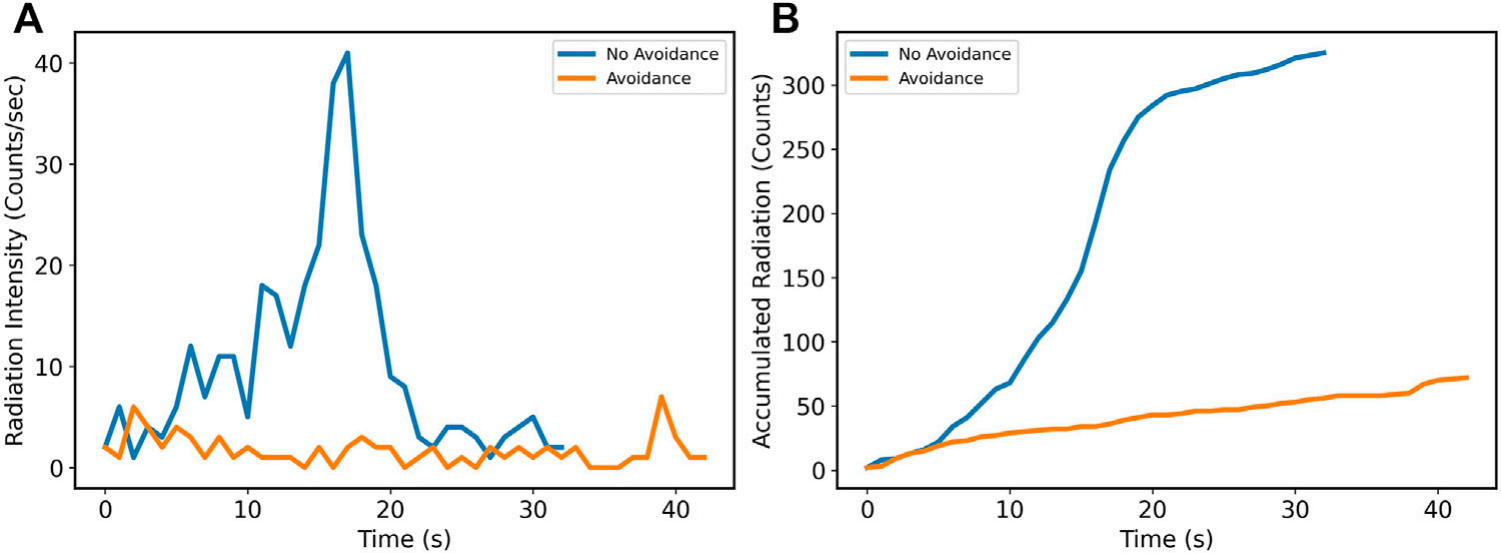
Radiation dose rate as a function of time **(A)** and accumulated dose **(B)**. The radiation dose increases when the robot passes in front of the radiation source when not avoiding radiation.

Figure 8 shows the difference in dose rates and accumulated dose measured by the on-board detector. Part 1) shows the count rate, with a clear increase as the robot passes the source, this results in a much greater accumulated dose, as shown in part (b). The accumulated dose (total recorded counts during navigation) for the enabled and disabled cases was 72 and 325, respectively, with a reduction by a factor of approximately 4.5 and the heavily shielded container providing a significant reduction in dose rate along the longer path. Effective shielding provides a route at similar rates to background; therefore, dose reduction can be extended indefinitely if the robot has an alternative path away from high-intensity sources. This demonstrates the utility of this approach to reduce radiation exposure to real-world robots operating in the presence of real-world radiation sources.

## Discussion

### Tuning of Navigation

For radiation avoidance to work effectively, there are steps when tuning a robotic system that can improve performance. These spatiotemporal factors are associated with radiation sensor update rate, robot velocity, and configuration space interpolation of radiation observations. The radiation sensor update rate dictates how often the radiation costmap is updated; for this discussion, a rate of 1 Hz is assumed for simplicity.

First, the velocity of the robot should be limited so as not to cover a significant distance before a new radiation observation is made. The term “significant” is in reference to two factors. Gradients in a radiation field can increase quickly due to an inverse square relationship of intensity with distance. Therefore, a robot travelling quickly can enter a high-radiation-field region with little prior warning. Furthermore, shielding materials may mask the presence of radiation sources until the robot is clear of these obstructions. If a robot covers more distance than the interpolation footprint, individual radiation observations will not be correctly averaged with other observations, resulting in a poor representation of the radiation field. It was found that linear velocities below 1 m/s are appropriate as a compromise between the spatial dimensions of the robot and, therefore, the interpolation distance whilst covering ground in a reasonable time, but this should be altered based on sensor update rate. Velocities that are too slow, though beneficial for radiation interpolation, leave a robot incapable of responding to threats in a timely manner.

Second, both the interpolation region of interest around the robot and the smoothing parameter *s* can have an impact on the accuracy of the reconstruction. It is suggested that the interpolation distance be larger than the product of the radiation sensor sample period and robot linear velocity. For example, a sample rate of 1 Hz is a period of 1 s, and a default Clearpath Jackal linear velocity of 0.4 m/s yields a distance of 0.4 m. A radius of between 0.5 and 1.0 m was used during the testing in this work. The smoothing parameter should be of scale lengths closer to that of the robot chassis itself; in this work, a value of 0.15 m was used.

Finally, the path planner of choice and the monolithic costmap must be able to update faster than the update rate of the radiation sensor; i.e., they can respond accordingly to new information. It is advised to update the path planner at least five times more frequently than the sensor update rate, as was used in this work. When using generic ROS path planners, it is advised to use radiation awareness primarily with a global planner for convenience. When using a radiation-aware local planner, it may need to be retuned to allow for enough deviation from the global path to avoid radiation. Furthermore, it is ensured the local and global planners allow for variable values of cost in their planning, not only lethal obstacles. The local and global planners used in this work were *Timed Elastic Band* ^4^ and *Global Planner* ^5^, respectively.

### Sensor Placement

As discussed previously, range sensors such as lidars provide a robot with the capability to effectively predict future costs based on its current velocity and the costmap that is generated from the lidar scan. By placing radiation sensors forward of the robot navigation centre (for ROS, this is typically known as *base_link* frame^6^), it grants an asymmetry biased to future positions the robot may be in. As costmap cells are on the order of centimetre scale, placing the sensor forward grants a few additional cells of forward planning for a path planner. The benefit is that the robot is much more likely to retreat away from radiation sources earlier in an unknown scenario.

For the experiments in this work, the radiation sensor was placed either at the navigation centre, as was the case for the single source, or placed slightly ahead of the robot navigation centre. In the case of real-world deployment at the Lancaster University Neutron Laboratory, only 10 cm, for frontier exploration simulation, this was 20 cm of forward displacement. Given a costmap resolution of 5 cm, this yields a few extra cells of planning range compared to the navigation frame.

For both the real-world and autonomous exploration experiments, the sensor was placed off-axis to the left or right side of the chassis. This offset leads to the robot’s having a preference whether to head left or right after encountering a source. This greatly decreases any oscillation in replanned paths, and it was found to be more reliable at repeatedly probing and eventually skirting around sources. Use of multiple sensors left and right would confer better interpolation and more robust behaviour.

### Variable Speed

This work assumes a constant linear velocity to simplify demonstration both mathematically and experimentally of how this approach can reduce radiation exposure, primarily through increasing the distance between the robot and ionising sources. Risk mitigation strategies such as ALARP/ALARA also leverage time as a dimension, as well as space, to reduce exposure for humans.

By increasing the speed of the robot in regions of increased dose, less time is spent in its vicinity, decreasing total exposure for the same manoeuvre. When twinned with operating at an increased distance as presented in this work, it is possible to further reduce total exposure. Variable speed is not advised for unknown environments, as it may negatively impact interpolation of the radiation field as previously discussed. Moreover, it may have unintended consequences on robot motion; for example, the robot is exposed to a occluded source and accelerates into the region of high radiation before path planning has accounted for the new information.

In known environments where there are no alternative routes, minimising time spent in view of an ionising radiation source in a predictable manner can further increase robot lifetime, with some possible penalties to other mission criteria such as battery life, which would otherwise limit nominal velocities.

## Conclusion

By having mobile robots practice radiation safety principles like their human counterparts, accumulated ionising radiation dose can be reduced, therefore increasing the operational lifetime of the robot before damage to components or materials leads to faults. The use of layered costmaps allows for risk from ionising radiation to be introduced into path planning and navigation approaches, with scalable thresholding to tune the response to this threat. By interpolating point observations into the configuration space of the robot, it is possible to avoid regions of high-intensity radiation and reduce accumulated dose significantly. In real-world trials at the Lancaster University Neutron Laboratory, rates were reduced to background levels, leading to a reduction in dose of approximately ×4.5, with simulations indicating an order of magnitude reduction is readily possible. This approach is applicable to unknown environments and for autonomous activities such as exploration, with the capability to adjust the response to radiation in real time.

## Data Availability

The datasets presented in this study can be found in the online Figshare repositories https://doi.org/10.48420/c.5801636 and the code developed is available in the GitHub repository https://github.com/EEEManchester/radiation_layer.

## Footnotes

1
http://wiki.ros.org/costmap_2d.

2
http://wiki.ros.org/move_base.

3
https://wiki.ros.org/gmapping.

4
http://wiki.ros.org/teb_local_planner

5
http://wiki.ros.org/global_planner.

6
https://www.ros.org/reps/rep-0105.html.
